# Generalized granuloma annulare associated with lung adenocarcinoma: a case report

**DOI:** 10.3389/fmed.2026.1662631

**Published:** 2026-05-08

**Authors:** Yanli Cai, Hongqu Tang

**Affiliations:** 1 Department of Dermatology and Aesthetic Medicine, Chongqing Hospital of Traditional Chinese Medicine, Chongqing, China; 2 Department of Oncology, Chongqing Hospital of Traditional Chinese Medicine, Chongqing, China

**Keywords:** case report, generalized granuloma annulare, lung adenocarcinoma, malignancy-associated granuloma annulare, paraneoplastic granuloma annulare

## Abstract

We report a case of generalized granuloma annulare (GGA) associated with lung adenocarcinoma. An old woman presented with generalized papules and plaques accompanied by pruritus that persisted for 1 year. Dermatological examination revealed densely distributed annular papules ranging from millet- to mung bean-sized lesions on the head, neck, trunk, and extremities. The majority of papules showed central umbilication, and some lesions coalesced into plaques ranging from pale red to dark red. Histopathological examination revealed epidermal hyperkeratosis with mild acanthosis. The superficial to mid-dermis showed a palisading histiocytic infiltrate between collagen bundles. Alcian blue staining was weakly positive (±), and crystal violet staining was negative. Endobronchial ultrasound-guided transbronchial needle aspiration (EBUS-TBNA) of the right lung revealed scattered clusters of atypical cells consistent with adenocarcinoma.

## 1 Case summary

We report the case of a 78-year-old woman who presented with generalized papules and plaques accompanied by pruritus for 1 year. The lesions initially appeared without obvious precipitating factors. Dermatological examination showed densely distributed annular papules ranging from millet- to mung bean-sized lesions on the head, neck, trunk, and extremities. The majority of papules had central umbilication. Some lesions coalesced into plaques and were accompanied by pruritus. No ulceration, exudation, or crusting was noted. Systemic symptoms such as fever or chills were absent. Initially, the patient was diagnosed at a local hospital with dermatitis/eczema and treated with anti-inflammatory and anti-allergic medications without significant improvement.

**Figure 1:**
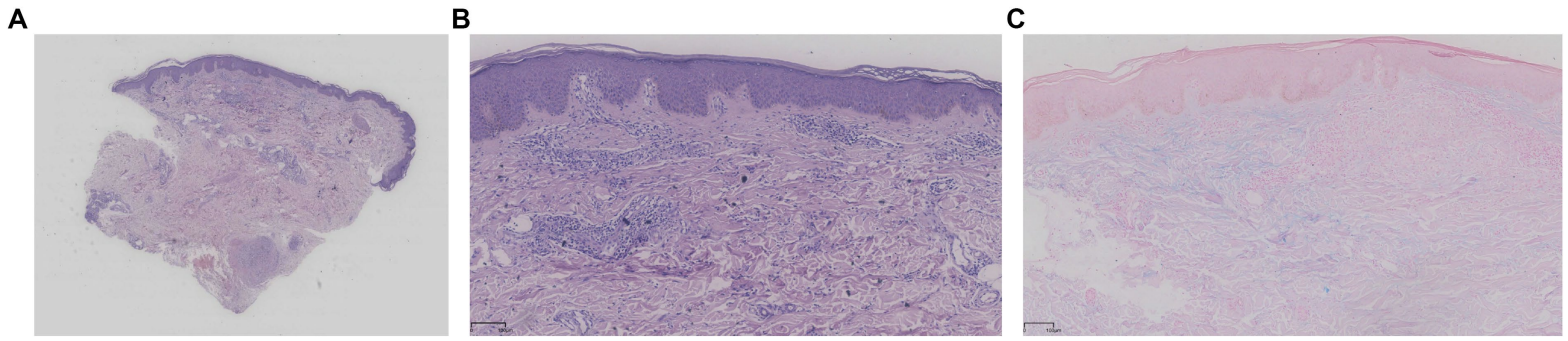
**(A–C)** Histopathological findings. Skin biopsy showed epidermal hyperkeratosis with mild acanthosis. A palisading histiocytic infiltrate was observed between collagen bundles in the superficial to mid-dermis. Alcian blue staining was weakly positive (±), and crystal violet staining was negative.

Subsequently, the patient presented to our hospital. A skin biopsy revealed epidermal hyperkeratosis with mild acanthosis. Histopathology showed a palisading histiocytic infiltrate between collagen bundles in the superficial to mid-dermis. Alcian blue staining was weakly positive (±), and crystal violet staining was negative (Figure 1). The patient’s general condition was fair, with normal appetite, sleep, bowel, and urinary habits, and no significant weight changes were observed. Her past medical history was unremarkable.

**Figure 2:**
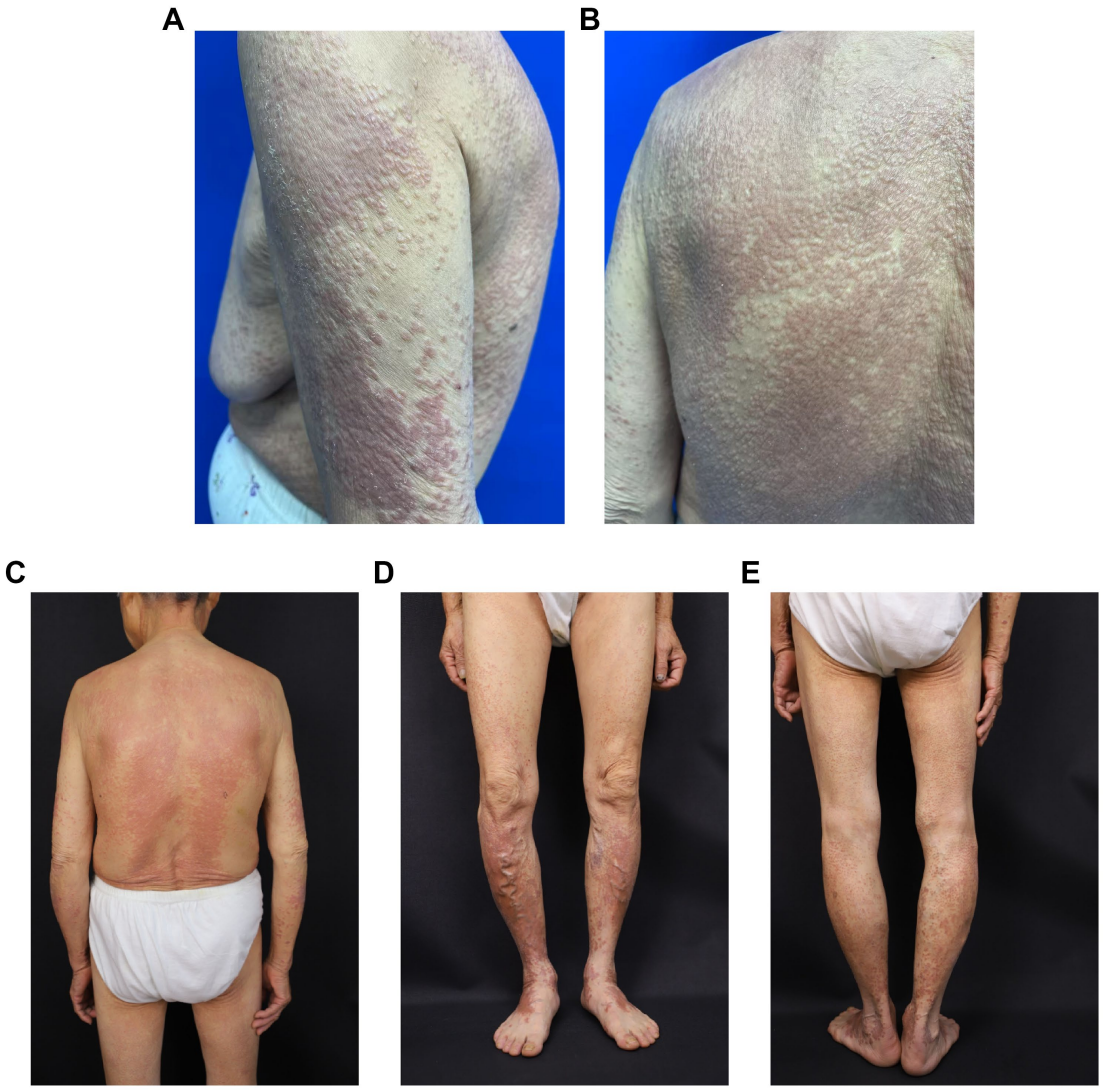
**(A–E)** Clinical photographs of skin lesions. Dermatological examination: Numerous densely distributed annular papules, ranging from millet- to mung bean-size lesions, were observed on the head and neck, trunk, and extremities. The majority of papules exhibited central umbilication, and some lesions coalesced into plaques. The lesions were pale red to dark red, without ulceration, exudation, or crusting.

Physical examination revealed stable vital signs and was otherwise unremarkable. Dermatological examination confirmed the presence of densely distributed annular papules ranging from millet- to mung bean-sized lesions on the head, neck, trunk, and extremities. The majority of papules had central umbilication, and some had merged into plaques with colors ranging from pale red to dark red. No ulceration, exudation, or crusting was observed (Figure 2).

**Figure 3:**
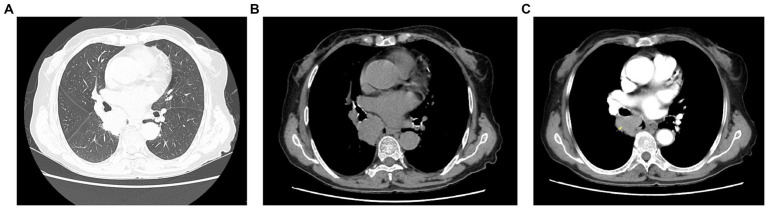
**(A–C)** Imaging findings. A 40.7 × 36.6-mm mildly lobulated soft-tissue mass was identified in the dorsal segment of the right lower lobe, with partial bronchial obstruction. Contrast enhancement was heterogeneous, and the lesion partially encased the right lower pulmonary artery.

Laboratory tests, including blood biochemistry, complete blood count, thyroid function tests, glycated hemoglobin, and serologic tests (syphilis, hepatitis C virus antibody, hepatitis B panel, and human immunodeficiency virus [HIV]), were normal. Contrast-enhanced chest computed tomography (CT) identified a mass in the dorsal segment of the right lower lobe. The mass was suspicious for malignancy and measured approximately 40.7 × 36.6 mm, with mildly lobulated margins; bronchoscopy was recommended. (2) Multiple scattered solid nodules were observed in both lungs (Figure 3).

**Figure 4:**
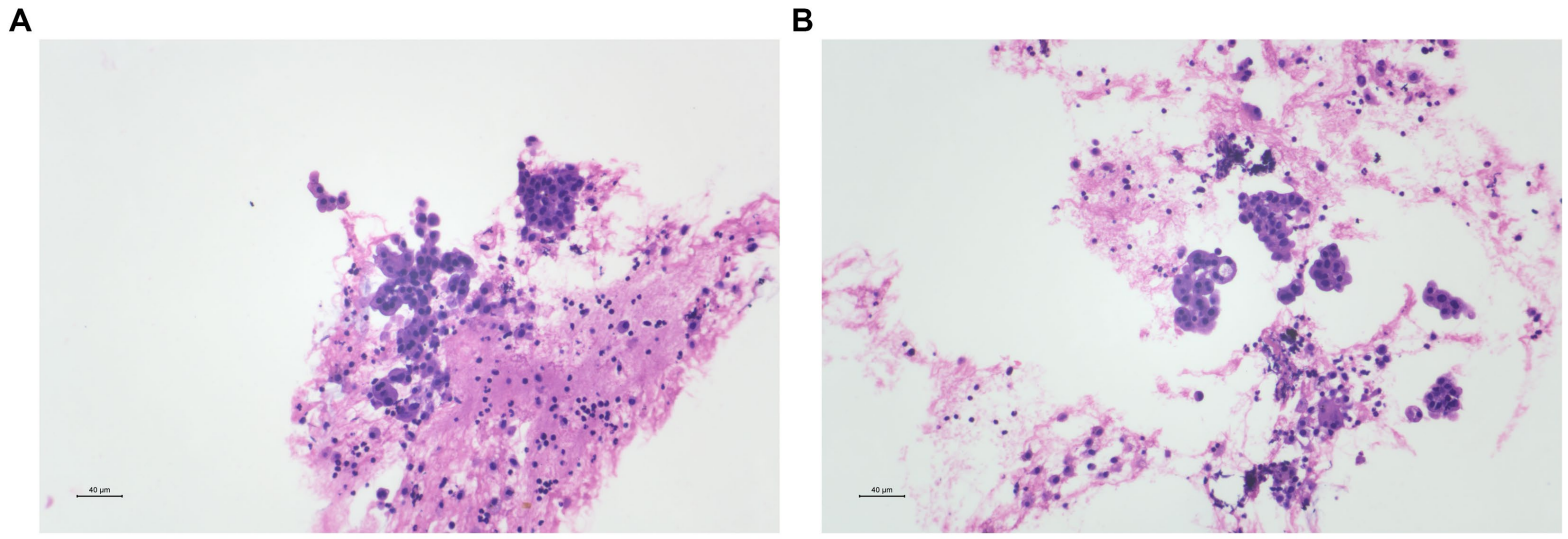
**(A,B)** Pulmonary histopathological findings. Endobronchial ultrasound-guided transbronchial needle aspiration (EBUS-TBNA) of the right lung revealed scattered clusters of atypical cells, consistent with adenocarcinoma.

**Figure 5:**
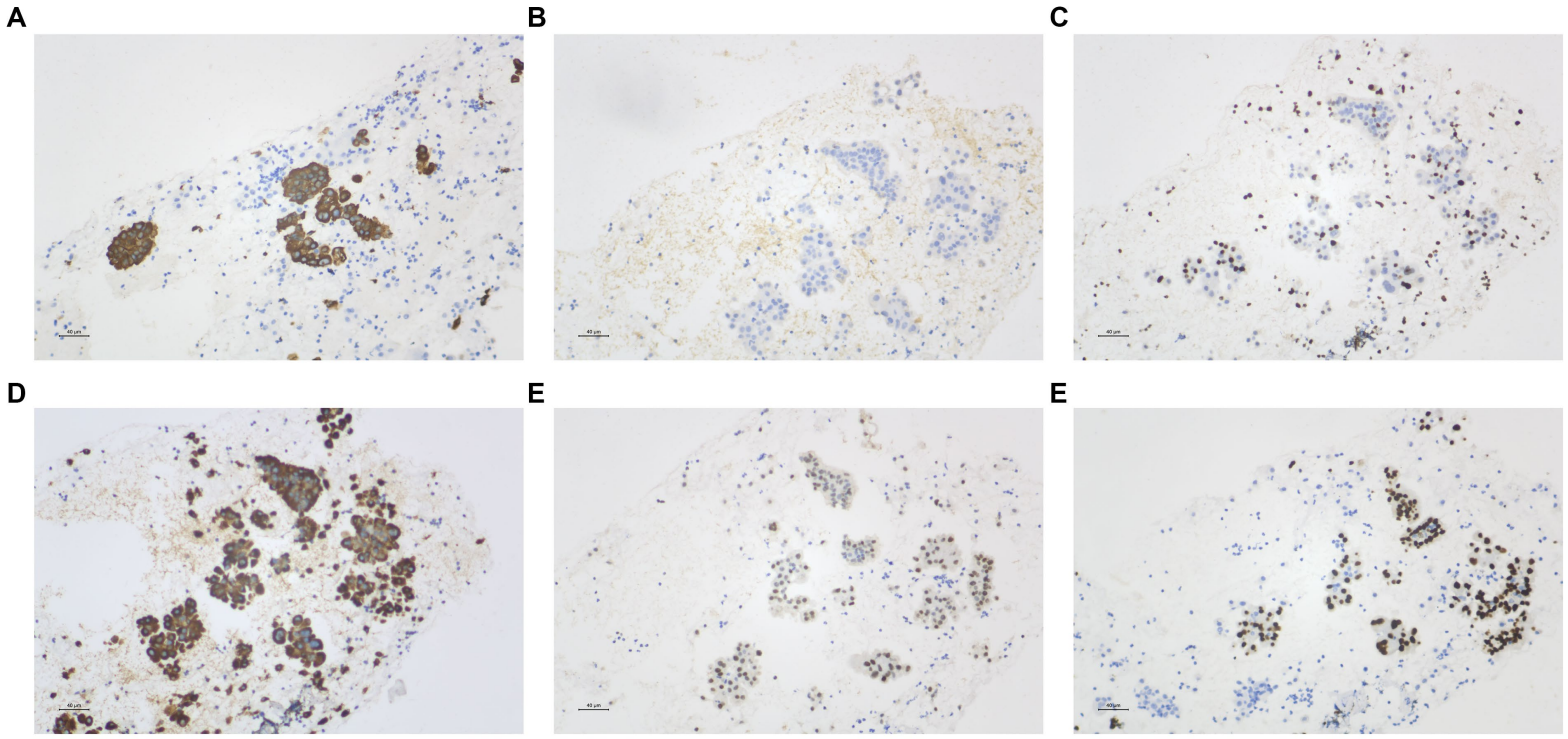
Immunohistochemistry findings. Immunohistochemistry showed: **(A)** CK (+); **(B)** CK5/6 (−); **(C)** Ki-67 approximately 5% (+); **(D)** p53 scattered positivity (+); **(E)** Napsin A (+); **(F)** TTF-1 (+). Notes: CK, Cytokeratin; CK5/6, Cytokeratin 5/6; Ki-67, Marker of Proliferation Ki-67; p53, Tumor Protein p53; TTF-1, Thyroid Transcription Factor-1.

Cytological examination of right intermediate bronchial puncture fluid revealed no malignant cells, with a few multinucleated giant cells present. Cytological analysis of right intermediate bronchial fine-needle aspiration smears also showed no evidence of malignancy. However, histopathological examination following endobronchial ultrasound-guided transbronchial needle aspiration (EBUS-TBNA) of the right lung showed scattered clusters of atypical cells, consistent with adenocarcinoma (Figure 4). Immunohistochemistry showed positivity for TTF-1, CK, and Napsin A, focal positivity for p53, negativity for CK 5/6, and a Ki-67 proliferation index of approximately 5% (Figure 5).

### 1.1 Diagnosis

The patient was diagnosed with generalized granuloma annulare and lung adenocarcinoma.

### 1.2 Treatment

Due to significant pruritus, the patient was treated with olopatadine and ebastine tablets for anti-allergic and antipruritic effects. Topical halometasone cream combined with vitamin E cream was applied under occlusion to provide anti-inflammatory effects, moisturize the skin, and promote lesion regression. Following treatment, pruritus was relieved, lesion coloration became slightly lighter, and some lesions became thinner and flatter.

Given the suspicion of generalized granuloma annulare (GGA), a chest CT was performed after the initial visit to screen for potential underlying malignancy. The examination revealed a mass in the dorsal segment of the right lower lobe, raising concern for a neoplastic lesion. Consequently, contrast-enhanced chest CT and endobronchial ultrasound were performed. Based on these findings, lung adenocarcinoma was definitively diagnosed. Considering the possible association between GGA and malignancy, no additional systemic dermatologic therapies, including systemic corticosteroids, hydroxychloroquine, biologic agents, small-molecule inhibitors, or photochemotherapy, were initiated. The patient was advised to promptly seek oncologic evaluation and treatment.

**Figure 6:**
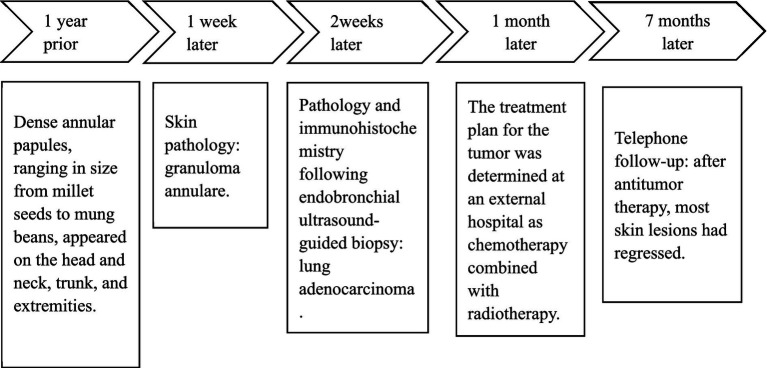
Timeline of events from initial skin lesion detection to definitive dermatologic diagnosis, tumor detection, tumor diagnosis confirmation, tumor treatment, and prognosis.

During the follow-up, the patient did not undergo surgical intervention and received chemotherapy and radiotherapy. After three cycles of chemotherapy (pemetrexed disodium combined with carboplatin), the granuloma annulare lesions showed a gradual regression trend during the course of lung adenocarcinoma treatment. Timeline of events is presented in Figure 6.

## 2 Discussion

GGA in adults is a chronic, benign inflammatory dermatosis. It presents as multiple annular or arciform papules and plaques, usually symmetrically distributed on the trunk and extremities. Clinically and histopathologically, GA has five subtypes: localized, generalized, patch (macular), subcutaneous, and perforating forms (1, 2).

Histologically, GA features collagen degeneration, palisading granulomas, and mucin deposition (3, 4). Focal collagen degeneration in the upper dermis is surrounded by palisading histiocytes, lymphocytes, and occasional multinucleated giant cells, forming palisading granulomas. Mucin deposition within degenerated collagen areas is typically highlighted by Alcian blue or colloidal iron staining. Immunohistochemical studies (5) have reported that infiltrating cells are predominantly CD4 + T cells, suggesting a Th1-mediated immune response. Occasionally, IgM and C3 deposits are detectable along vessel walls or at the dermoepidermal junction, indicating possible immune complex-mediated mechanisms.

The exact cause of GA remains unclear. Current research has indicated potential associations with immune dysfunction, genetic susceptibility, metabolic disturbances, infections, and malignancies. Studies have reported that adult generalized GA may coexist with acute myeloid leukemia, other malignant tumors, metabolic abnormalities, thyroid disease, interstitial lung disease, essential thrombocythemia, autoimmune disorders, and HIV infection (6, 7). Its pathogenesis might involve the degeneration of collagen in the dermal and subcutaneous layers, possibly driven by an immune-mediated delayed hypersensitivity reaction, with tumor-associated immune factors further influencing this mechanism (8).

In this case, imaging identified a mass in the lung, and EBUS-TBNA combined with immunohistochemical analysis confirmed lung adenocarcinoma. Tumor-associated antigens may activate a Th1-driven inflammatory process, leading to GA as a skin manifestation of systemic antitumor immune responses. Previous studies have reported that (9) GA patients exhibit an elevated risk of hematologic malignancies. Among solid tumors, GA has been most commonly associated with prostate cancer and gastrointestinal malignancies (10). In addition, certain biologic agents used in cancer therapy, such as tumor necrosis factor-*α* (TNF-α) inhibitors and interleukin inhibitors, have occasionally been reported as potential triggers of GA (11). These observations suggest that the tumor itself may contribute to GA development through immune-mediated mechanisms.

In a large-scale statistical cohort study, Barbieri et al. included 5,137 patients with GA and 51,169 patients with benign skin diseases (diagnosed as nevi or seborrheic keratosis). The two cohorts were well-matched, and no statistically significant difference was found in malignancy incidence between them. This finding suggested that GA might not be associated with an increased risk of solid-organ malignancies (12). Conversely, Bagci B presented a different perspective based on statistical analysis, indicating a closer relationship between GA and malignancy with increasing patient age. These cases of GA demonstrated resistance to conventional therapy but showed resolution following treatment of the associated malignancy, suggesting an underlying relationship (13).

Currently, GA associated with malignancy is termed malignancy-associated granuloma annulare (MGA) in the literature and classified as a paraneoplastic syndrome. MGA does not significantly differ morphologically from typical GA lesions, often manifesting as generalized distributions. MGA lesions have a higher incidence and greater intensity of pruritus and closely correlate with tumor status. Histopathologically, MGA lesions are similar to typical GA, characterized by collagen degeneration, palisading granulomas, and mucin deposition. However, differences may be observed in the morphology and distribution of inflammatory cells and the severity of perivascular inflammation.

At present, literature addressing the association between lung cancer and GA is limited, mostly consisting of case reports. Recent reports (14) have suggested that MGA predominantly occurs in female patients, typically aged between 59 and 69 years. The time interval between the onset of GA skin lesions and tumor diagnosis remains unclear. In terms of treatment, MGA lesions typically resolve following surgical removal or effective management of the primary tumor (15). Consistent with these reports, our patient was a female who was diagnosed with lung adenocarcinoma approximately 1 year after the onset of generalized GA. Following three cycles of chemotherapy, the tumor significantly decreased in size, accompanied by regression of skin lesions. Therefore, when encountering paraneoplastic GA, the clinical priority should be placed on identifying and treating underlying malignancies.

In summary, the relationship between GA and malignancy has not been definitively established. Current evidence is largely derived from case reports and observational studies, which suggest a possible association but do not demonstrate causality. Although GA is generally considered a benign reactive inflammatory condition, clinicians should remain vigilant for underlying hematologic or solid malignancies in selected patients. Well-designed prospective studies are required to further clarify the potential link between GA and cancer.

## Data Availability

The original contributions presented in the study are included in the article/supplementary material, further inquiries can be directed to the corresponding author.
